# Eco-decisional well-being networks as a tool for community decision support

**DOI:** 10.3389/fevo.2024.1210154

**Published:** 2024-04-18

**Authors:** R. S. Fulford, E. Paulukonis

**Affiliations:** 1 Office of Research and Development, US Environmental Protection Agency, Gulf Breeze, FL, United States,; 2 Office of Research and Development, US Environmental Protection Agency, Research Triangle Park, NC, United States

**Keywords:** ecosystem services, human well-being, networks, community decision making, network indices

## Abstract

Community decision making based on the sustainability of ecosystem services is an integrated process that involves multiple complex decisions and is greatly aided by an understanding of how those decisions are interrelated. The interrelatedness of decisions can be understood and even measured based on connections between actions and services and influence of services on domains of human well-being. These connections can be formed into a network structure so that quantifiable properties of networks can be applied to understanding decision impacts. We developed an eco-decisional network based on weighted social-ecological networks as a tool for integrated decision making based on ecosystem services and human well-being. Nodes are actions, services, or domains of human well-being and they are linked by weighted influence derived from community stakeholder input. Examination of the eco-decisional network, as well as comparison to pattern in the random networks, suggest there are important patterns of influence among different influence pathways from actions to community well-being, which describe community priorities and define unique roles through which chosen sets of actions can influence human well-being. The eco-decisional network is generalized across communities but can also be made community specific, which provides a tool for comparison between communities in decisional priorities (network properties), as well as comparisons between proposed actions within a community (network paths). The well-studied properties of networks, well-established network theory, as well as established network metrics make this approach promising for application to integrated decision making and for communicating possible outcomes to stakeholders. The result is a guidance tool for connecting propose actions to ecosystem services and human well-being.

## 1 Introduction

Community decision making concerning natural resources and infrastructure is a complex issue that affects stakeholders through gain or loss of ecosystem services (Rutherford et al., 2018; Pouso et al., 2020). Ecosystem services are direct benefits to people from nature such as extractable resources, clean air and water, recreational opportunities, and viewscapes (Costanza et al., 1997). These services, alongside social and economic services can inform complex decisions if they can be effectively measured, and requisite decision trade-offs communicated to stakeholders (Bingham et al., 1995). The challenge lies in integration across services as most complex decisions affect multiple services at once and usually represent trade-offs as potential actions yield different integrated results. Sustainability is best understood as a whole system trait rather than through management of multiple single-issue criteria (Bodini, 2012). Many tools and strategies have been employed to identify integrated outcomes and even qualitatively rank them in terms of predicted impacts on services (Poch et al., 2004; Martin et al., 2009). However, here we are interested in quantitative options for understanding the integrated connections between proposed actions and service outcomes and argue that services-based decision making has common properties with network-based flow analysis that may be useful for understanding integrated service outcomes. Decision makers need to identify quantitative endpoints like human well-being as the outcome of all decisions made that affect all stakeholder types as a community (Costanza et al., 1997), and human wellbeing can be meaningfully used in network-based analyses of decisional outcomes.

Network tools describe connections (links) between groups of objects (nodes) that have individual identity but also form a collective whole defined by how strongly and completely the nodes are interconnected. Network tools have been used to describe social groups such as community health networks (Manning et al., 2014), energy groups such as food webs (Niquil et al., 1999), and informational/influence groups such as ecosystems (Raffaelli, 2006; Fiscus, 2009). In turn, network flows can describe connectivity but can also be used to quantify relative connection strength and even flow of materials through the network between nodes of interest to inform system robustness (Patricio et al., 2006; Jorgensen and Ulanowicz, 2009; Ulanowicz, 2009). In its simplest form, network analyses have been used to predict group organization and assembly in terms of how links are formed between nodes (Butts, 2008), but in cases where links can be weighted or quantified, network analysis allows for understanding of organization in terms of relative link strength (Zorach and Ulanowicz, 2003) and even total network throughput (Huang and Ulanowicz, 2014; Ulanowicz et al., 2014). The latter has given rise to a suite of descriptive network indices based on optimal levels of organization (Ulanowicz, 2009). Combined these network analyses have been used to explore common ground in network organization and to quantify network pathways as a method for understanding the effects of change on the network as a whole.

Here we introduce the idea of an eco-decisional network, as a derivative of social/ecological networks, in which the nodes are actions and outcomes rather than physical elements, such as in a social network connecting people. We could assemble a decisional network that linked together decision makers and stakeholders as nodes, but this would create a focus on how decisions are made, not the cause and effect of those decisions, which is our focus here. The challenge of this novel approach is to define nodes in a meaningful way and here we employ the ecosystem services concept and define nodes as either actions to be taken, services affected by these actions, or the resultant impact of change in service production on human well-being. To avoid an open-ended dichotomy, we employ a suite of action categories (Fulford et al., 2017), service categories (Fulford et al., 2015; Summers et al., 2016), and domains of human well-being (Summers et al., 2014) that have been well studied and defined based on analysis of real-world decisions. In this framework, a decision is a pathway from an action through affected services to a domain of human well-being and the network as a whole describes the decision space for a particular group of stakeholders (e.g., community) that predicts the collective impact of multiple decisions on human well-being. Our interest is in identifying the relative importance of different pathways and possibly exploiting network theory to identify useful patterns across all pathways in the network. By using an established framework for both node definition and linkages, we are tying this analysis to existing decisional science (Diener and Seligman, 2004), but also making progress in their use through the organization and analysis of network properties.

Eco-decisional networks are a hybrid concept in network analysis in that links can be weighted based on influence but flow between nodes is diverse and not conserved, so not directly comparable as in a network with a single common currency of throughput. This places a limit on the use of tools that focus on a conserved flow though the network (e.g., Ulanowicz et al., 2014). However, advances have been made in our understanding of weighted socio-ecological networks (Zorach and Ulanowicz, 2003; Dykstra et al., 2016) that provide some useful quantitative tools for constructing and analyzing decisional networks and perhaps finding some common ground for comparative study. We apply these tools to the development of the eco-decisional network and explore general and community-specific patterns in network properties as an introduction to this approach for decision support. The goal is to demonstrate tools grounded in network theory that can be used by community decision makers to support integrated decision making across multiple complex issues and be transferable to multiple communities.

## 2 Methods

### 2.1 Eco-decisional network assembly

Networks are comprised of nodes connected by links. In the case of our eco-decisional network, nodes are actions and outcomes of decisions, and links are the level of influence decisions have on services or the influence a change in services have on domains of human wellbeing. Flow influence is expressed as flow weights (sensu Zorach and Ulanowicz, 2003) and provide the basis for a network analysis of overall influence in the form of path weightings and network indices of overall information content (Ulanowicz, 2009). Again, pathways from action to well-being nodes represent potential decision options so we will explore network metrics that focus on different path choices and their relative influence on human well-being as an endpoint. First, we describe network assembly by defining node types and link weighting. This is followed by a random network analysis to look for patterns in network size and link weight distribution and for comparison to our real-world network as well as similar previous work (Zorach and Ulanowicz, 2003). We then assemble a community-specific network analysis based on community input from structured decision-making exercises in multiple index communities (described below) and place the results in the context of observed pattern as a way of generalizing eco-decisional networks across communities.

Community network elements (nodes and links) were taken from three sources and connected based on community specific data on links and weights. Human well-being (HWB) was defined using the human well-being index (Summers et al., 2014), which is based on a set of eight HWB domains (Table 1). Human well-being nodes were the outcome of planned decisions and are comprised of eight HWB domains connected to a node for the overall HWBI. Service nodes represented the initial effect of planned decisions and were defined with a set of 22 nodes defined based on analysis of connections between ecological, social, and economic services and HWB (Summers et al., 2016). These services were linked to decisions through a set of 29 action categories define based on a series of facilitated workshops conducted in nine communities across the country (Fulford et al., 2016) combined with a keyword analysis of an additional 97 community planning documents (Fulford et al., 2017). The workshops were designed to elicit information on action priorities important to each community, as well as priorities and connections among these action priorities, defined services, and the domains of human well-being.

### 2.2 Eco-decisional network description

Nodes in ecological and social networks are typically physical elements of the real world such as species, individuals, populations, physical locations, or objects of confluence (e.g, computer network nodes). These are useful when the network flow is left unmeasured or can be directly measured as physical transfer of energy, carbon, etc. Our decisional network measures flow as ‘effect’ which can come in multiple physical forms with the common outcome of altering benefits to people. The nodes in our eco-decisional network are either decision outcomes (actions), measurable benefits (services), or domains of HWB (domains). We also include a final node for an index of overall human well-being (HWBI) which is an optional upper endpoint of network flow (Table 1).

#### *Action categories* (AC; n=29) –

These are nodes describing potential actions to be taken as a result of community decision making. Such actions can take many forms such as physical (e.g., build roads), financial (e.g., economic development), or legal (e.g., protect historic buildings). The Action category nodes are designed to capture a broad and comprehensive suite of potential actions which are organized into categories to maximize transferability among communities. The categories were assembled based on workshop outcomes describing action priorities combined with a keyword analyses of community planning documents. Data on specific action priorities were assembled into action category nodes based on expert opinion.

#### *Service categories* (S; n=22)–

Actions yield impacts primarily as changes in services to community stakeholders. The Service category nodes describe benefit outcomes in three broad areas (environmental, social, and economic). These nodes are prescribed by well-being theory and there are 22 Service nodes used in this analysis which were described by Summers et al. (2016).

#### *Domains of human well-being* (D; n=8) –

These nodes describe how service benefits to stakeholders translate to a change in human well-being. There are eight domains of human well-being as described in the HWBI overall structure and these domains create a path for a decision to affect HWBI in a measurable way (Summers et al., 2014).

Human well-being Index (HWBI; n=1) – The HWBI node is the measurable index of human well-being. This node is included as the integrated endpoint of the decisional network and allows for the domain nodes to have differing influences on overall well-being as defined by link weights.

#### Link weighting –

**FIGURE 1:**
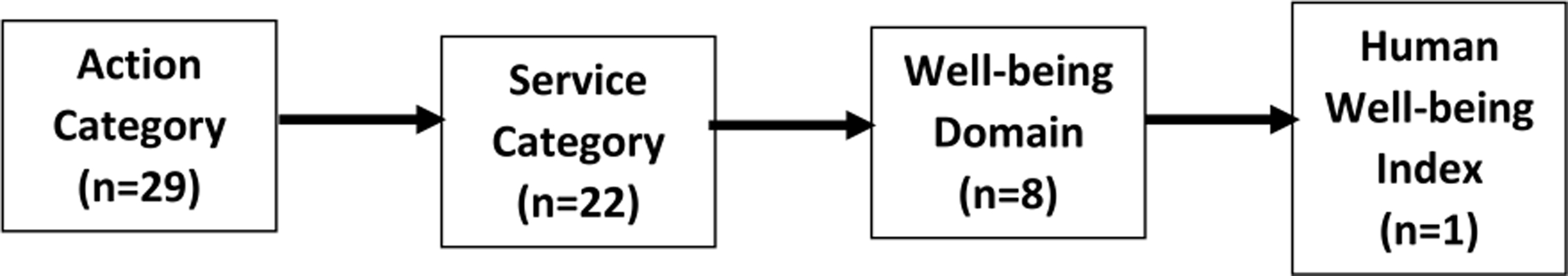
Lindemann spine diagram of decisional well-being network showing direction of influence flow from Action category nodes to the index of human well-being. Arrows do not indicate link weights between individual nodes.

Links are connections between nodes and can be defined based on measurable weight/flow, as well as directionality of flow (unidirectional/bidirectional). For instance, a social network is usually defined as unweighted and bidirectional in that connections are all equivalent and effect nodes mutually. In contrast, an ecological network may consider differences in relative flow between particular nodes and that flow may also be directional in that flow is measured from node A to node B but not from node B back to node A. The decisional network is a hybrid social/ecological network that does not measure physical flow between nodes. Alternatively, we consider differences in flow weights as the effect of a change in the source node on change in the receiver node. These weights are directional and represent the strength of influence a decision has on a service, a service has on a domain of human well-being, or the influence of individual domains on the HWBI (Figure 1). Decisions are pathways from actions to well-being so the cumulative weight of the links making up a pathway represent its relative importance to the overall decision space. The eco-decisional network does not allow for flow in the opposite direction, nor does it consider cross flow within node type, such as influence of service nodes on each other. The latter is an important subject but not considered here. Weights can fall to zero effectively removing the influence between nodes. Weights are normalized influence scores (0–1) assigned individually to each link in the network (See below).

**FIGURE 2:**
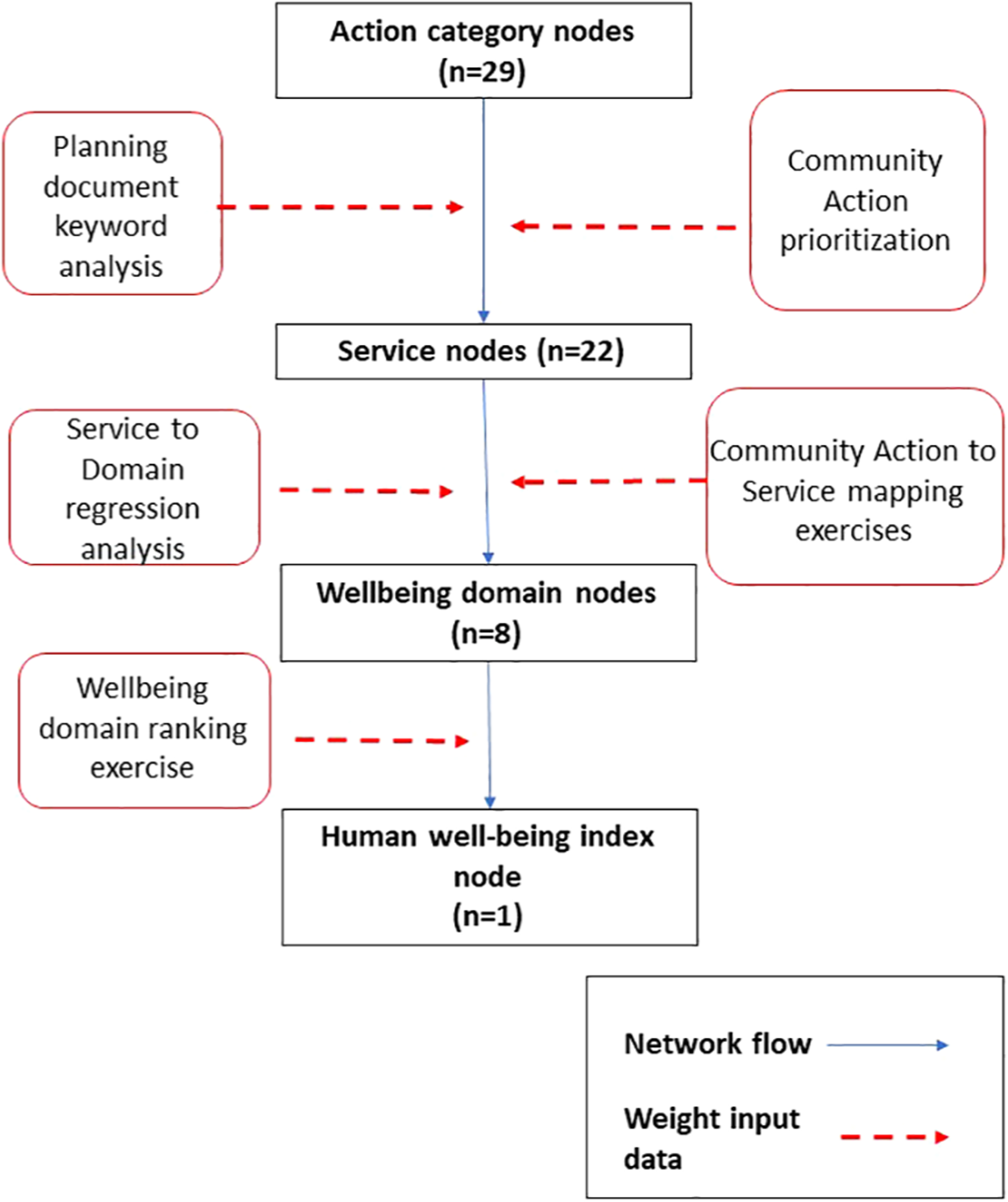
Flowchart summarizing determination of weights for node links by link type. Planning document keyword analysis was described in Fulford et al. (2017). Community action prioritization, Community actions to Services mapping exercises, and Well-being domain ranking exercises were conducted during stakeholder engagement described in the Supplementary Data section. All data were normalized (0–1) within link type for inclusion in the network analysis.

Link weights for the community specific eco-decisional networks were assigned to node and link types described above based on numerical output from workshop discussions (Figure 2; n=9) and the related keyword analysis (Fulford et al., 2017). Weights represent the cumulative results of relative importance estimates derived from group discussions and anonymous prioritization exercises (Community Comparison Report; https://cfpub.epa.gov/si/si_public_record_report.cfm?Lab=NHEERL&direntryid=330853), as well as direct count of how often connections were mentioned in analyzed documents (Fulford et al., 2017). All raw weight scores were normalized (0–1) within type (e.g., keyword counts) and consolidated across data types for each specific node-node link. Full details on weight derivations can be found in the Supplementary Data. Link weights for the random network analysis were assigned randomly (0–1) within network size which was defined by node count.

### 2.3 Measures of weighted network properties

Network properties can be described by comparison of individual paths (cumulative weight) through the network or using indices of overall network organization. For path comparisons, the focus is on cumulative connection strength between select action categories (potential decisions) and domains of human wellbeing (decision outcomes) with services acting as intermediary effects. Comparing paths can be as simple as relative weights for two alternative paths (which action is more influential on a specific well-being domain) or a more complex comparison examining relative effects of chosen actions on all domains of human wellbeing (decision trade off comparison across all domains). Network level indices describe overall organization, and the literature reports a suite of possibilities including those with origins in exergy (Jorgensen and Ulanowicz, 2009) and diversity (Ulanowicz et al., 2009) indices. Here we focus on the latter and apply a set of indices described by Zorach and Ulanowicz (2003) for weighted networks to analyze relative linkage weights to describe the balance between network connectivity and mutual path information. These indices are particularly useful for decision making as Zorach and Ulanowicz (2003) described how relations between network connectivity and number of realized roles (i.e., pathways) can be used to optimize flow across an entire network, and we use this concept here for maximization of influence on community well-being as a function of resource investment.

#### *Ascendancy* (A) –

Ascendancy has been called the scaled mutual constraint (Ulanowicz et al., 2009) of a network and is generally the information content of a network (e.g., what does knowledge of node *i* tell us about node *j*)? in that higher Ascendancy indicates more network efficiency and therefore more dominant (higher weighted) connections. However, ascendancy has an upper limit or capacity, and as A approaches capacity, the network becomes less flexible and more sensitive to perturbation. In the case of the eco-decisional network this upper limit would be viewed as a dilution of influence or excess redundancy. Network capacity is considered fixed for eco-decisional networks as we describe them here.

#### *Connectivity* –

This index is the weighted average of links per node which describes how connected the nodes are as a whole and includes both presence and importance of connections. Link counts per node vary between 0 and N-1 each with a weight between 0 and 1. Therefore connectivity (C) is a measure of both network size and organization. In an examination of natural ecological systems, Ulanowicz et al. (2009) described a window of vitality (WOV) in ecological networks for which connectivity ranged from approximately 1 to 3.01 for these ‘natural’ networks. Increasing connectivity increases redundancy in the network and reduces importance of individual paths.

#### Realized network roles –

Roles are unique sets of paths through the network which can be interpreted as choices for achieving specific outcomes. A role can be shared completely or partially across multiple decisions pathways in that they each have similar influence on human wellbeing. This means that the number of realized roles (R) will increase with network size but also decrease with increased redundancy among pathways. An understanding of this redundancy across the whole network describes the amount of mutualism (Fath, 2007) across available pathways and how this redundancy affects network robustness (Fath, 2015). This metric describes how efficiently available resources like time and funds are being allocated to achieve objectives integrated into human well-being. The number of realized unique roles in a network are impacted by both link density and relative link weight in that large differences in weight will create more unique roles. Ulanowicz et al. (2009) in their analysis of natural systems reported their WOV had a range for realized roles between 2 and 4.5 across multiple networks. There is a balance between diversity of path options and redundancy that defines the overall information content of the network captured by network indices.

### 2.4 Random network analysis

Random network analysis was used to examine patterns in the relationship between described network-based indices and both network size and complexity. This approach builds on the random network analysis of Zorach and Ulanowicz (2003) and with a comparison of our network space to their ‘window of vitality’ describing the proposed bounds of connectivity and information content of ecological networks. Network size is defined based on combined value of node number and number and weight of existing links. Complexity was defined based on network indices of mean connectivity (C), Ascendancy (A), and Realized Roles (R) to measure patterns with network size and complexity and compare the outcome for our random networks to reported values from the literature as a tool for understanding observed patterns that can aid in defining the most efficient and robust structure for an eco-decisional network. We examined a random group of 500 networks ranging from n=20–100 nodes and from 0 to an upper limit of *n x n* links. The weight of each defined link was also randomly set between 0 and 1. The values of C, R, and A were examined across the range of sizes and compared to similar output reported by Zorach and Ulanowicz (2003). Comparisons were made graphically to published data and to examine relationships among indices associated with network size and complexity that can be used to interpret community-specific network data.

The random network analysis was conducted with three slightly different constraints reflecting three relationships with the community-specific eco-decisional network. The fully random network with varying size (i.e. node count) is described above and was used to describe the unconstrained pattern of network indices with size and to compare our results to those previously reported by Zorach and Ulanowicz (2003) in their ‘window of vitality’. The second random network analysis was based on the same 60 node set used in the community specific analysis (Table 1) but with randomly assigned link weights with two levels of constraint. The community random network analysis was first run with links limited to all possible AC-S, S-D, and D-HWBI links, as in the community specific network. Link weights were varied randomly between 0 and 1 allowing for a maximum possible link count of 822. The second level of constraint limited links to the set used in the community specific network (287; See Supplementary Information) but with weights set randomly (0–1). The community decisional random network results were compared to both the full random network results as well as the community specific network results described below as a bridge between values of the network indices for the unconstrained and the constrained network outcomes.

### 2.5 Real world network comparison

Community specific network analysis was based on community-defined links between the 60-node set defined in Table 1. An eco-decisional network was built based on community engagement data defining weights (0–1) for nodes representing action categories, services, and domains of wellbeing. Link weights were assigned based on community data for relative importance (n=60; l ≤ 822; See Supplementary Information). This community specific network iteration represents the real-world decision space for an amalgamated community seeking to achieve improvements in stakeholder well-being by replacing a ‘one decision at a time’ approach with an integrated decision option.

#### Node and Edge selection and weighting –

A series of workshops combined with a keyword analysis of community planning documents were previously described and used for both node identification and weighting in the community-specific network development. Edge weights were derived differently for each edge type (AC-S; S-D; D-HWBI).

Action category (AC) nodes represent potential actions or decisions to be made by a community seeking to improve human well-being as a path to increased sustainability. Action category nodes were identified by workshop participants in nine separate communities, compiled into an overall list, and combined with similar keyword-based results from 97 community planning documents. This list of specific actions was further consolidated into action categories based on an expert discussion of the original action list. The resulting action categories were used as nodes in the community specific eco-decisional network in order to maximize transferability across communities.

**FIGURE 3:**
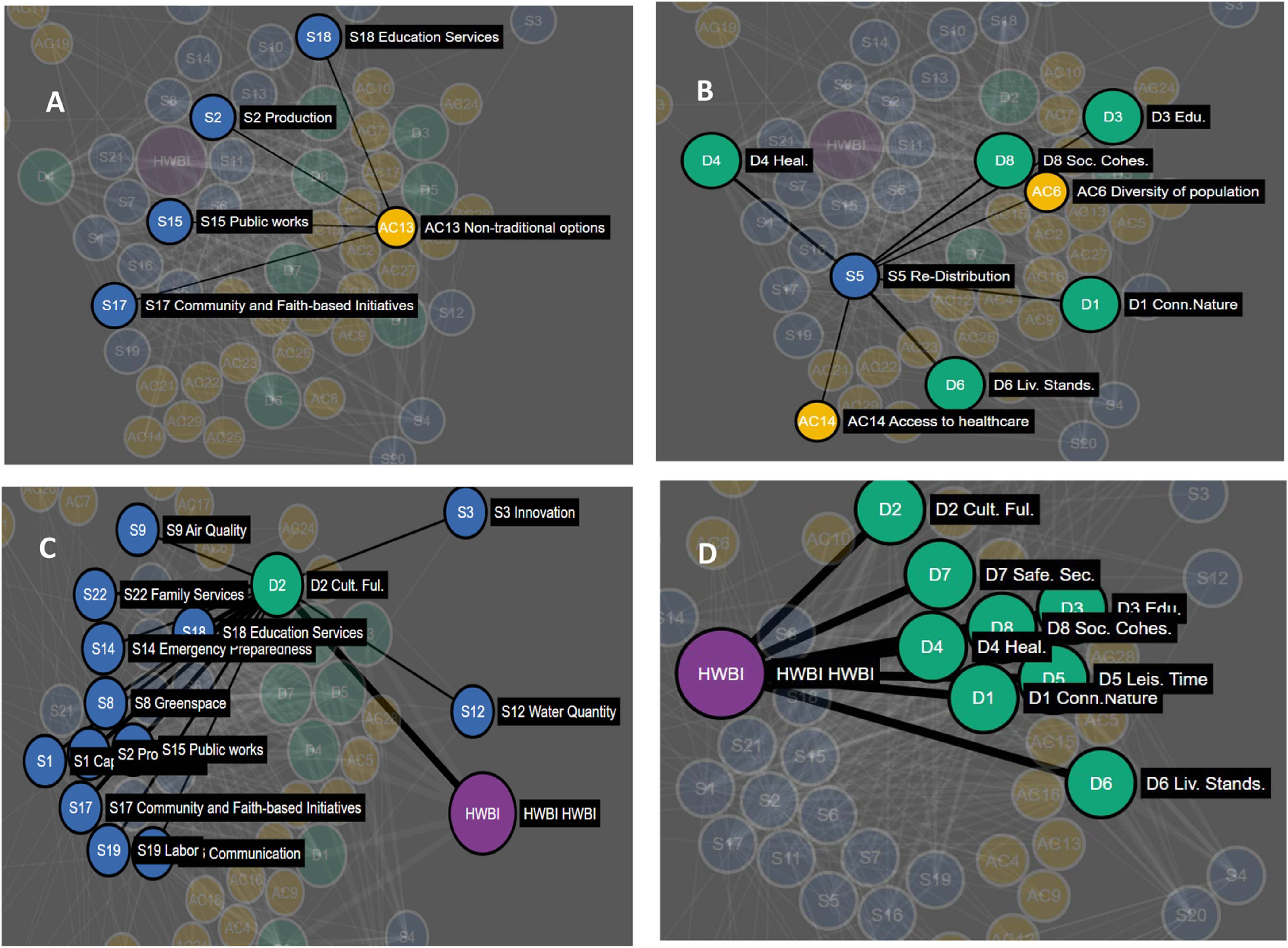
Decisional network diagram highlighting **(A)** Action category (yellow) to Service (blue) links. Example AC-S link shown is Non-traditional options linked to four service nodes: Community and Faith-based initiatives, Public works, Production, and Education services. Decisional network diagram highlighting **(B)** Service links to both Action category and Domains of human well-being (green). Example AC-S-D link shown is the Re-distribution service node linked to Action categories and Domains of human well-being. Decisional network diagram highlighting **(C)** Service nodes links to Domain nodes and the Human well-being Index node (purple). Example Domain of human well-being is D2. Cultural Fulfillment. Decisional network diagram highlighting **(D)** Domain node links to the Human well-being Index node (HWBI; purple). All Domain nodes are linked to the Human well-being index nodes as these are the eight Domains that combined to calculate HWBI. See Supplementary Information for full list of nodes by type.

Service nodes (S) were identified with a national study on the impacts of Services on Domains of human well-being (Summers et al., 2016). These service categories were used as nodes in the community specific eco-decisional network but also cross-referenced with workshop and keyword analysis results to identify AC-S links (Figure 3A). Weights for identified AC-S were normalized (0–1) from priority voting results conducted during workshops and counts of service mentions in planning documents.

Domain nodes (D) were set based on the eight domains of human well-being based on the formal development of HWBI as an overall index of human well-being (Summers et al., 2014). Formal prioritization of the domains of human well-being were conducted during community workshops (Fulford et al., 2016) to estimate community priorities and set weights for identified S-D and D-HWBI links in the community specific eco-decisional network (Figures 3B, C). The national HWBI study also applied a regression approach to quantify relationships between services and HWBI domains and these regression results were used to standardize S-D link weights based on the workshop outcomes into a cumulative weight for each S-D link in the network (Figure 3D).

All domain nodes were linked to HWBI but could have varying weights. Weights on links between domain nodes and the HWBI node were estimated based on community discussions combined with group voting activities during workshops, which were designed to place the eight domains of HWBI into priority order. The results of the ranking exercises were normalized (0–1) to the maximum value reported and combined across the communities to obtain a composite set of D-HWBI link weights, which were used as weights in the community specific eco-decisional network analysis.

### 2.6 Analysis of community specific network based on network indices

Methods for community comparisons were explored as a methodology for defining optimal network organization around realized roles (R) and mean connectivity (C). Random network iterations of link weights (0–1) represent the theoretical bounds and patterns of change for network indices similar to the ‘window of vitality’ described by Zorach and Ulanowicz (2003). These bounds and patterns were compared to the realized community network’s properties as a method for ranging differences among hypothetical communities.

## 3 Results

### 3.1 Random network analysis

**FIGURE 4:**
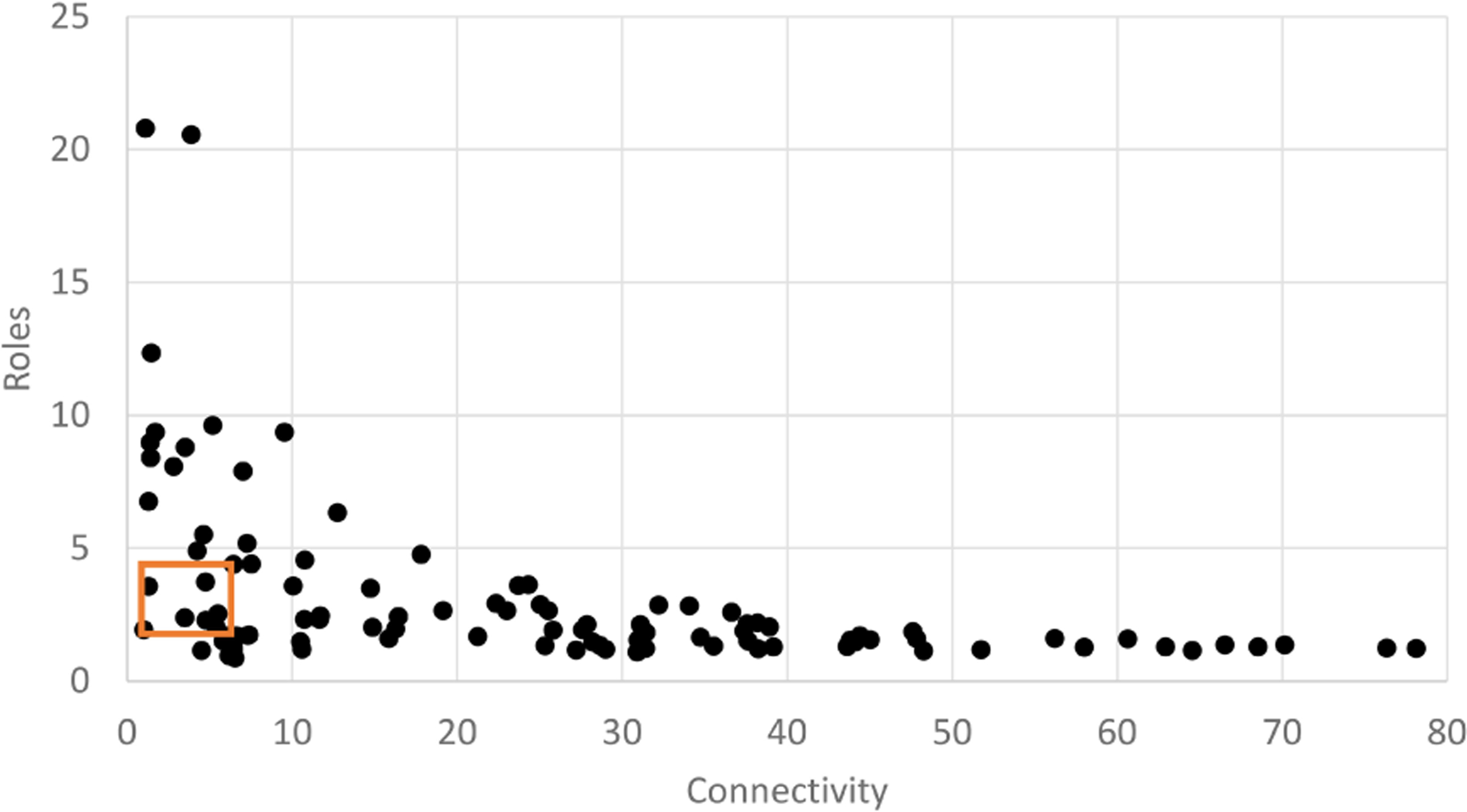
Scatter plot of mean connectivity (C) vs. realized roles (R) for a set of 500 random networks. Each network has a random node count (size; 0–100 nodes) and a random weight (0–1) for each link. Polygon shows ‘Window of Vitality’ described by Zorach and Ulanowicz, 2003, which brackets a set of real-world ecological networks.

A set of 500 networks with random size (n=20 to 100) and random link weights (0–1) were assembled and compared based on calculated values for the network indices (C, R, and A). The range of values were plotted and compared to published results for random networks in Zorach and Ulanowicz (2003). Random network results demonstrated a clear pattern between mean connectivity and realized roles with R highest at lowest connectivity and dropping rapidly with a cluster of values centered on the region referred to as the Window of Vitality (Figure 4). This demonstrates the pattern between R and originally described by Zorach and Ulanowicz (2003) for weighted networks. Pattern indicates relationship between C and R with unique roles in the network dropping as connectivity increases.

**FIGURE 5:**
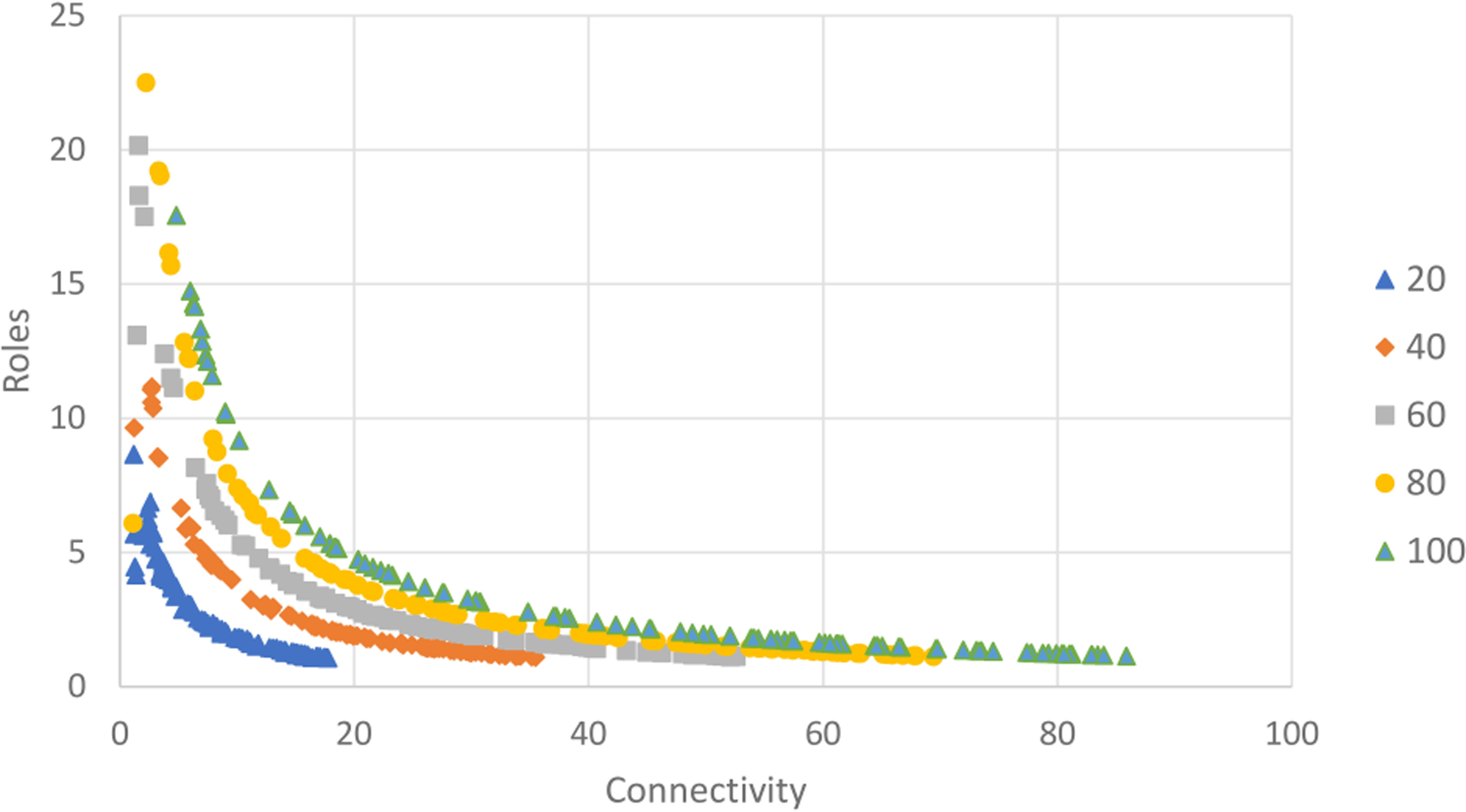
Scatter plot of mean connectivity (C) vs. realized roles (R) for a set of 500 random networks. Each network has a node count within a size group (100/group; node count = 20, 40, 60, 80, or 100) and a random weight (0–1) for each link. Data are plotted in size groups.

If we delineate random networks into size groups (n=20, 40, 60, 80, 100) and replot the pattern between C and R takes distinct shape indicating that as link density (C) increases with a fixed node count, the relationship between connectivity and realized roles can be well described with a power law function. Realized roles is maximized at low connectivity for a given network size and initially drops rapidly as connectivity increases but approaches a minimum value for R for a given network size (Figure 5). For a fixed node size, the pattern is very consistent.

**FIGURE 6:**
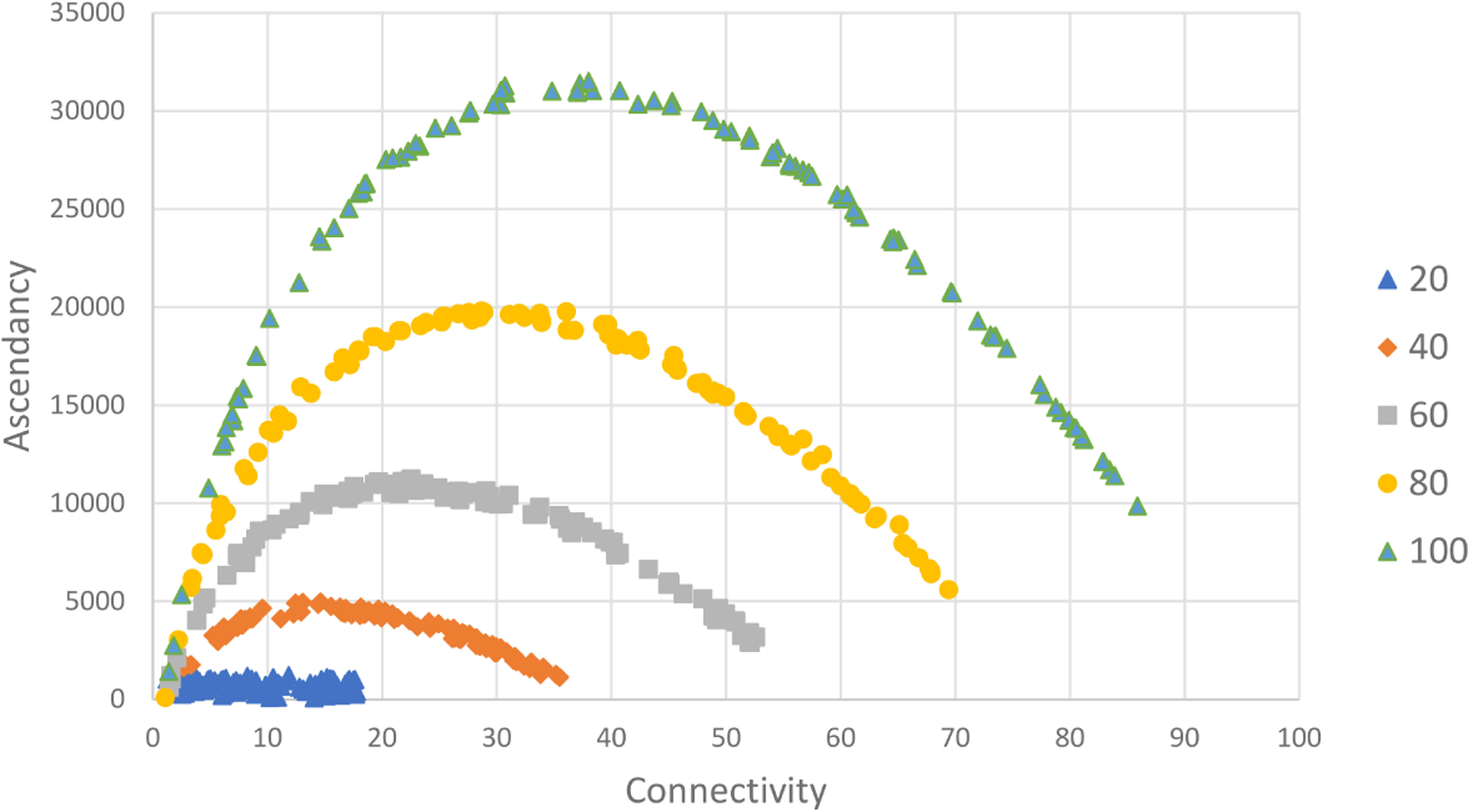
Scatter plot of network Ascendancy vs. Mean connectivity for 500 random networks delineated by network size (n=20–100) measured as number of nodes. Links randomly assigned weight between 0 and 1. See text for details.

The relationship between the mutual information index, ascendancy, and mean connectivity was slightly different. Pattern with network size is also evident for our index of network mutual information (A) (Figure 6). The Ascendancy index has a parabolic relationship with mean connectivity indicating that network information about the described system increases at low connectivity but reaches a maximum and begins to decline as network redundancy increases. For instance, in the network size group (n=40), the maximum for A is reached at a C value of approximately 14.8 which is consistent with the C value at which the decline in number of roles begins to slow (Figure 5). The pattern is clearer for Ascendancy, but the Realized Roles (R) is a more understandable index of network value as roles represent decision pathways from Actions to Well-being.

### 3.2 Community specific network analysis

**FIGURE 7:**
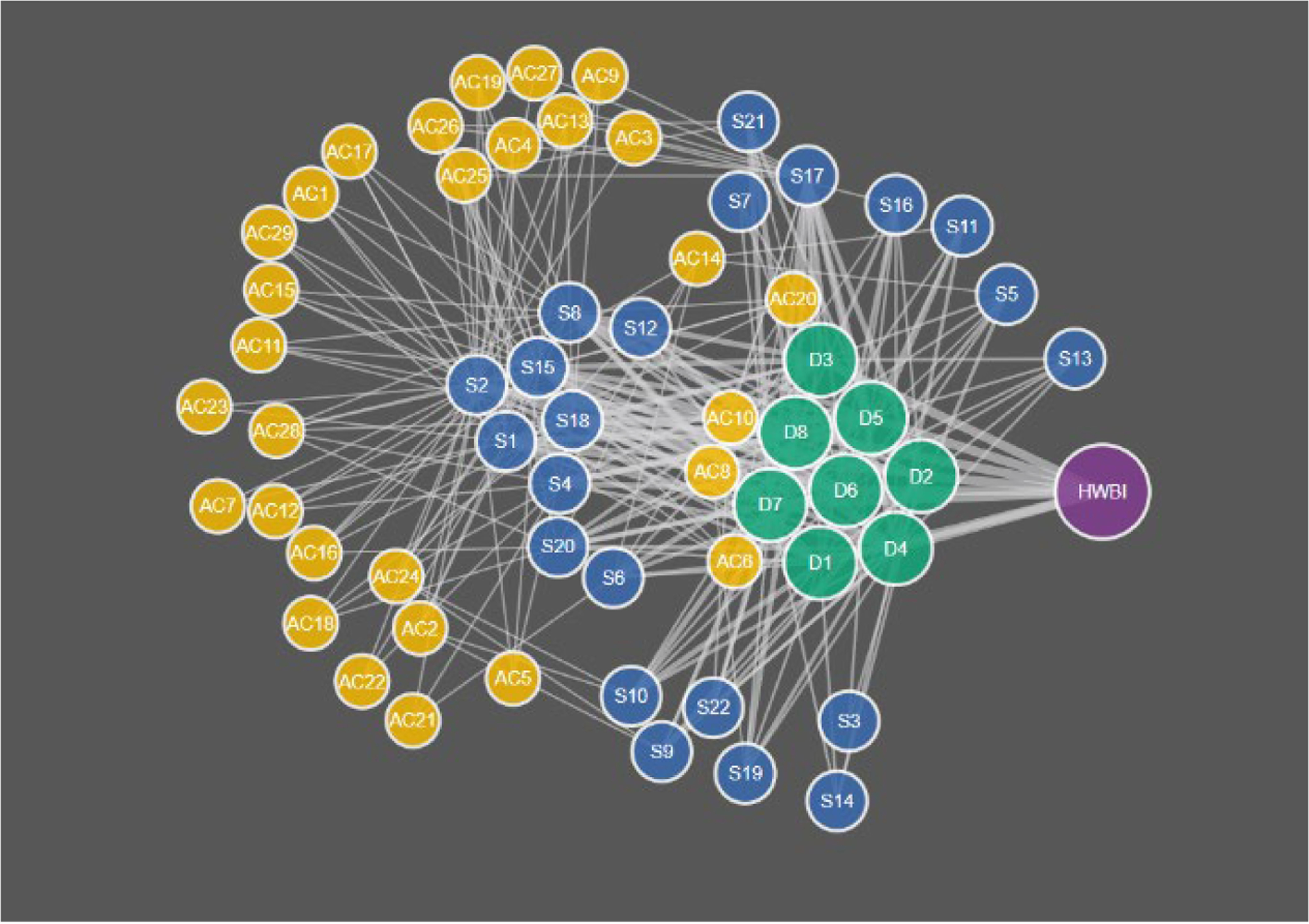
Diagram of whole community specific eco-decisional network structure including all nodes listed in Table 1 and Figure 1. Network nodes have four types: Action categories (yellow), Services (blue), Domains of human well-being (green), and HWBI index (purple).

The second part of the analysis involved homing in on the node/edge structure of the eco-decisional network. The eco-decisional network includes 60 total nodes in three types (Action category, Service, and well-being domain, HWBI; Figure 7; Table 1), and the random network analysis for the eco-wellbeing network included weights assigned to 822 possible links in the unconstrained random test (AC-S, S-D, and D-HWBI links only), and 287 possible links in the random test limited to community specific links only (links identified during community engagement only; See Supplementary Information).

**FIGURE 8:**
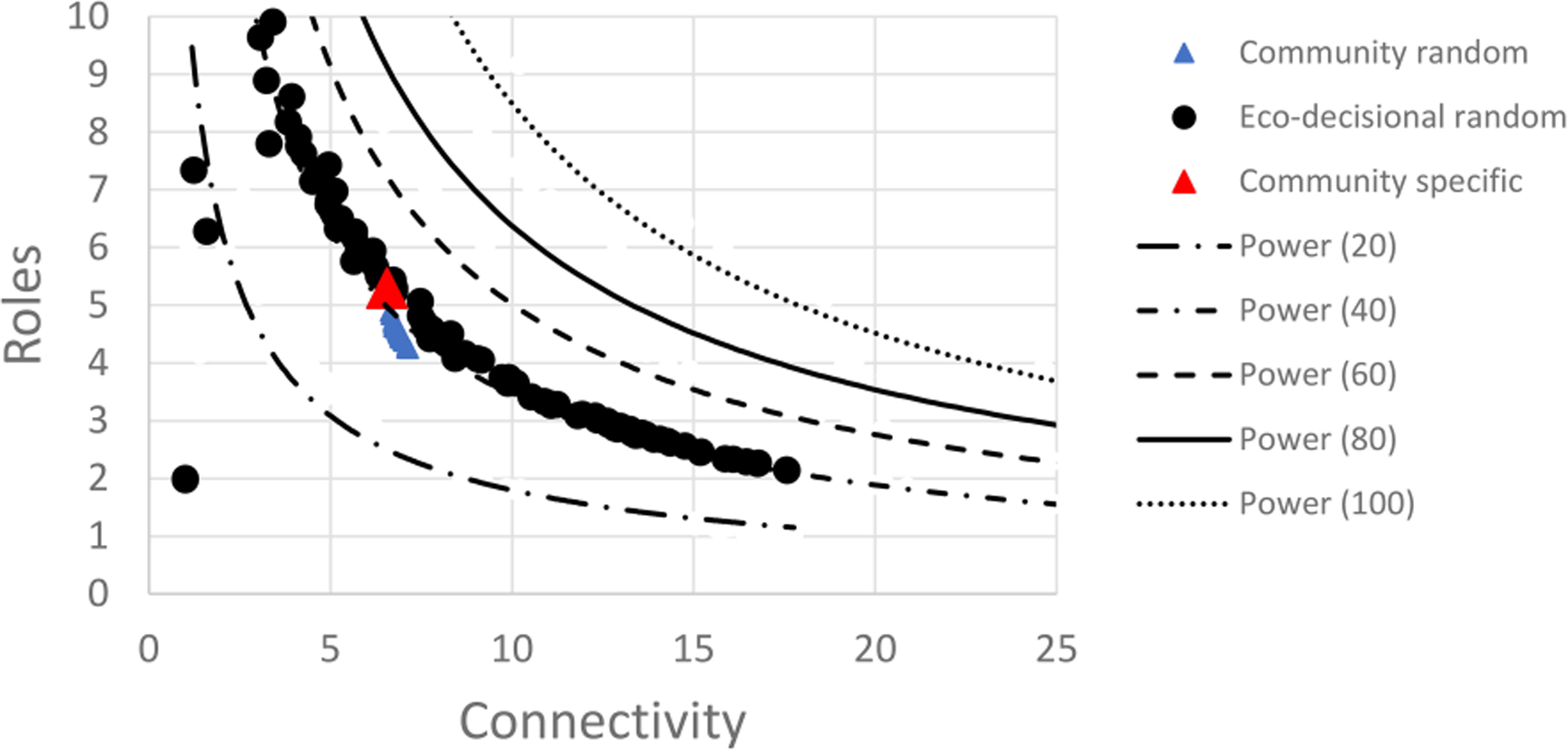
Scatter plot of random decisional network results for mean connectivity and realized roles showing power relationship for random network sizes between 20–100 nodes each (lines). Data points show distributions for 100 random eco-decisional networks with 64 nodes and either a maximum of potential links up to 822 (black circles), a restricted set of random links up to 287 (blue triangles), or the single community specific network with 64 nodes and 287 non-zero links (red triangle). See text for details.

**FIGURE 9:**
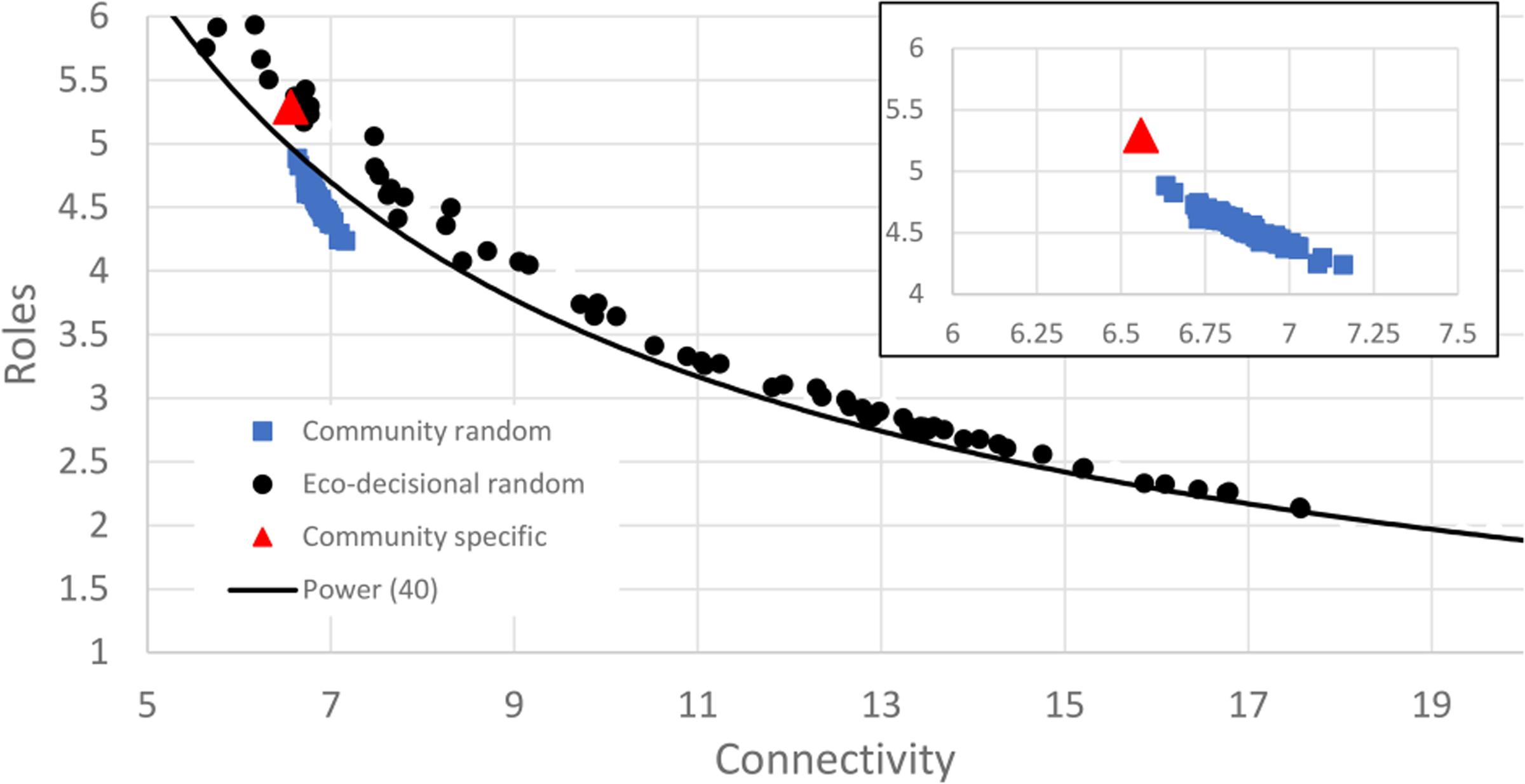
Scatter plot of random decisional network results for mean connectivity and realized roles showing power relationship for random network of size 40 for reference (black line). Data points show distributions for 100 random eco-decisional networks with 64 nodes and either a maximum of potential links up to 822 (black circles), a restricted set of random links up to 287 (blue triangles), or the single community specific network with 64 nodes and 287 non-zero links (red triangle). Inset figure shows eco-decisional data in more detail. See text for details.

When the eco-wellbeing random network was compared to the results of the full random network analysis, the 60 node eco-wellbeing network showed a similar pattern for Realized Roles vs. mean Connectivity but better resembled the smaller (n=40) network than the random network of similar size (Figure 8). The random analysis of the restricted eco-wellbeing network, which was limited to 60 nodes and 287 links in three categories showed a similar pattern but represented a small subset of the unrestricted community network near the middle of the R vs. C range (Figure 9). Finally, the community specific network containing 60 nodes and link weights based on specific community input generated an R vs C value near the top of the restricted network range consistent with the overall range for the random network results (Figure 9 inset).

The community specific network (Figure 9) had a connectivity of 6.7 links per node and 5.3 realized roles from 60 nodes and 287 links between nodes. The community data were positioned at the upper end of the C vs. R curve for community data suggesting this is a maximum number of unique roles and a minimum connectivity. Likely shifts in the decisional network made through changes in relative weight of decisional pathways would therefore reduce unique pathways between action categories and human well-being and increase network redundancy.

## 4 Discussion

Network tools and analyses have been used to identify optimal organizational patterns in ecological, social, and hybrid networks. The value of understanding network organization is that information flow through networks can be optimized to achieve complex goals such as ecological/economic stability (Cumming et al., 2014; Huang and Ulanowicz, 2014), optimality of information flow in social networks (Butts, 2008; Henry et al., 2014), how network indices may inform important concepts like sustainability (Opsahl et al., 2010; Hu et al., 2017), and as we sought here a balance between decisional impact and redundancy of actions for integrated decision making.

Decisional science is largely a social discipline and there is a robust body of research on network theory as it pertains to social organization (Manning et al., 2014; Dykstra et al., 2016). However, the emphasis has largely been on unweighted network analysis intended to elucidate organization (Opsahl et al., 2010) and the process of network assembly (Butts, 2008) as factors in understanding social interactions. In contrast, ecological network analysis builds on material flow theory (Ulanowicz, 2001), and is intended to understand networks as a suite of interrelated throughput pathways that vary in importance but collectively follow similar organizational constraints as social networks (Lau et al., 2017). In a review of network analyses, Lau et al. (2017) considered both social and ecological network analyses and observed that integration across disciplines was possible but required common use of definitions and tools for analysis. Zorach and Ulanowicz (2003) in an attempt to generalize network theory based on ecological throughput presented an approach to applying throughput calculations such as Ascendancy to weighted networks and explored observed patterns in network complexity. Here, we have expanded on this approach to consider network organization as a tool for informing community decision making, where we observe similar pattern in the balance of realized roles (e.g., pathways through the network) and network complexity.

**FIGURE 10:**
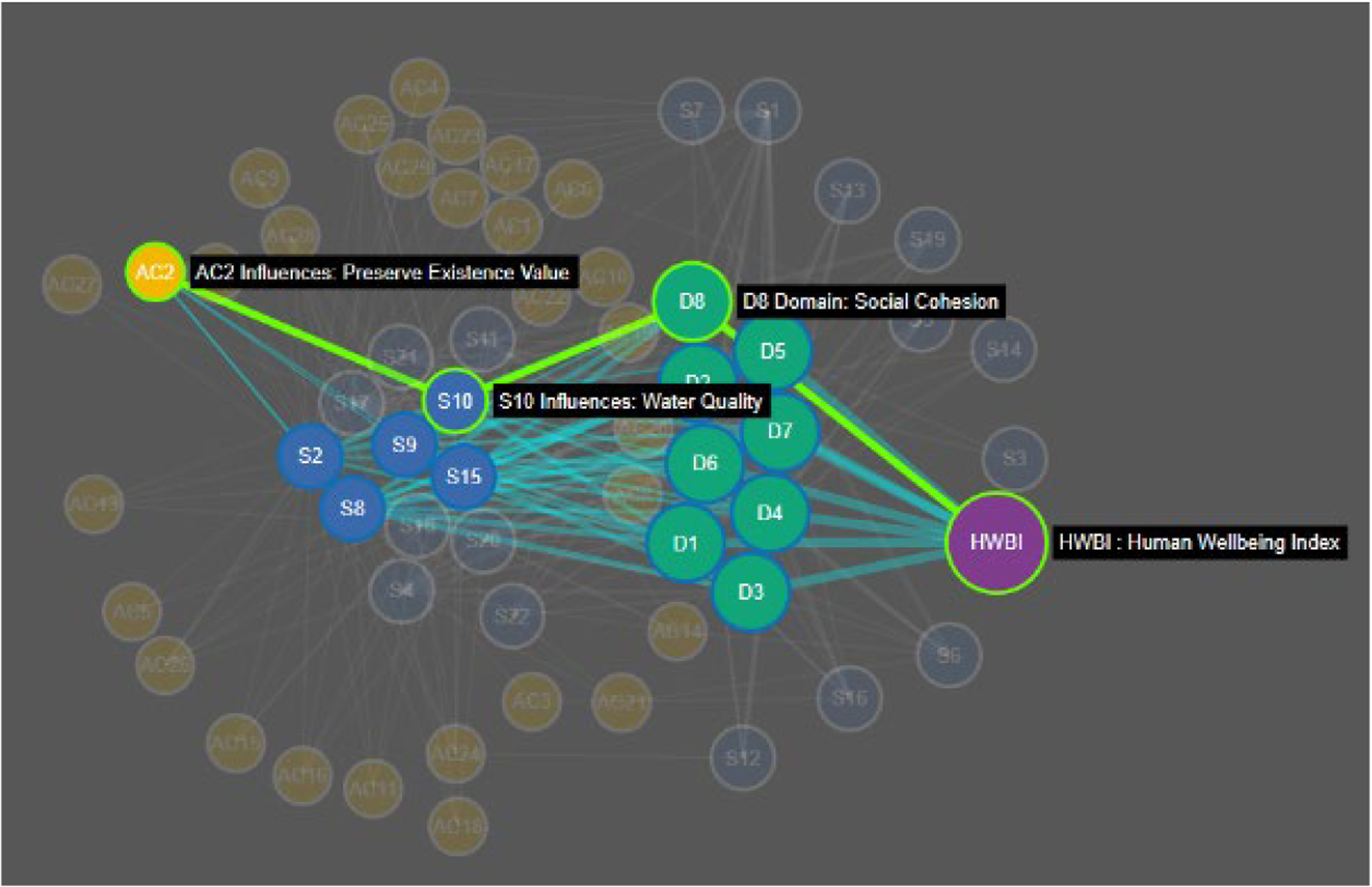
Example pathways analysis based on a community-specific eco-decisional network. Pathways are shown for connecting action category (Preserve existence value) to the index of Human well-being (HWBI) through affected service and HWB domains nodes. Bolded path is a theoretical chosen path (highest weight) for action considered in the context of all the other options.

Complex community decisions involve multiple potential actions and multiple paths to desired wellbeing outcomes. In our discussions with community stakeholders emphasis was placed on a shift from multiple independent decisions (e.g., economic development vs. public safety) towards an integrated approach that seeks common endpoints. In the example shown in Figure 10, there are multiple pathways from the Action Category (e.g., Preserve existence value of existing natural resources) to overall human well-being, but the most influential pathway passes through the Service ‘Water quality’ and the wellbeing domain ‘Community cohesion’. In this example existence value means clean water, which yields a sense of community to the stakeholders. This is useful information for forging a decision, but other such dominant pathways can also be identified across the entire network and used both to understand their collective effect on wellbeing, but also identify the number of dominant pathways (Roles). According to our network analysis there should be no more than 6 Roles and this number could be reduced as low as 3 to maximize efficiency of effect on human well-being. Communities can use the eco-decisional network tool to focus their actions into the most efficient overall structure. This process is made less complex if we can identify the descriptive network structure, and both intermediate and final outcomes that are well-defined and measurable. This demonstration of an eco-decisional network tool can aid decision makers in making this shift by identifying relative roles and most influential paths from action categories to HWB Domains. General patterns highlighted in the random network analysis indicate that changes in community priorities, quantified as network link weights, can influence the range of network indices, which follow a power law function between impact of individual decisions and the redundancy of impacts across different decision pathways. Further the link constraints of the eco-decisional network result in network indices comparable to unconstrained networks of a smaller size (node count). These network characteristics affect outcomes in a predictable way and are adjustable based on a communities choices. Ascendancy has been described as the amount of information contained in a network tool (Ulanowicz, 2002) and in this case information is the different ways a suite of potential decisions may result in desired outcomes. Decision support is driven by such information and our analysis indicates that a maximum can be reached based on optimal values for network connectivity and realized roles.

The eco-decisional network is also useful for understanding trade-offs among potential decisional pathways as all paths in the network are defined in a comparable manner. These trade-offs become important when community decisions are made across multiple pathways with limited resources. Trade-offs highlighted between connectivity and number of unique roles capture the choice between trying to do a lot at once vs. choosing the most direct path to specific goals. Shifts in realized roles reflect the level of integration among decisions that is impacted by the level of connectedness among decisions. Therefore, changes in C and R for a community-specific eco-decisional network that are driven by how Action-Service-Domain of well-being links are defined reflect real world outcomes. This is consistent with similar network-based community analyses of sustainability (Bodini, 2012). Our analysis shows that as connectivity increases the real number of paths (roles) in the network drops rapidly and the decision space shifts from high impact through fewer specific pathways to a diverse outcome that may have wide impact but requires investment in many more action categories at once to achieve a desired outcome. This pattern is consistent with the stability-complexity debate in ecosystem network theory (Haydon, 1994), which is called network robustness by Ulanowicz et al., 2009. In their analysis of ‘real-world’ ecological networks, Zorach and Ulanowicz (2003) highlighted the ‘Window of Vitality’ as a generalizable pattern in network assembly observed across different network assemblies. The window of vitality concept has been broadened to examine similar patterns in economic networks (Zisopoulos et al., 2022). In a similar manner we observed a generalizable pattern in our analysis of real-world networks in the form of a power law function. Since this is a decisional network there is no absolute optimum but rather a range of choices to be made based on community priorities. That said the Ascendancy value of our community-specific eco-decisional network was well below the predicted optimum based on random network analysis suggesting there is room for improvement in information content of the network before the cost of redundancy is maximized.

The eco-decisional network can also inform a direct comparison of decisional pathways. Pathway trade-offs in the community-specific eco-decisional network will help inform priority setting not just for specific actions but across multiple actions that may not seem strongly related when examined in isolation. As an example, the action category (Preserve existence value) might be chosen as a decision category of interest intended to protect natural capital (e.g., streams) as a desirable feature of the landscape to community stakeholders based purely on its existence. Such benefits are hard to define in isolation, but the network can be used to show the multiple pathways by which an investment in existence value can yield an increase in services that are tied to HWBI (Figure 9). Once pathways are known then choices can be made as to the desired path(s) (e.g., investment in stream water quality) based on available resources, circumstances for early action, and its perceived impact. Most importantly, the chosen path of action can be compared in the network to other options in terms of relative impact so that the decision is driven not just by predicted outcomes but also by the opportunity cost of other possible outcomes. The optimality and consensus achieved in network development give structure to these trade-off comparisons as well as a clear visual and numeric method of communication, which is important for stakeholder acceptance.

In the example community described in the Supplementary Data and the associated report (Deeper Look at Ouachita river: https://cfpub.epa.gov/si/si_public_file_download.cfm?p_download_id=542172&Lab=CEMM), four action categories were defined and mapped to domains of human wellbeing to allow for development of a community eco-decisional network. Prior to network development the relative importance of these four action categories in terms of their impact on community objectives was not quantified and any trade-off decisions among the respective pathways was not definable. The development of the network tool allowed for the relative roles and connectivity to be quantified so that the optimal pathway to well-being was identified as investment in greenspace and dredging of the river. The exercise also identified several potential shifts in network weightings possible through changes in how action categories were carried out that would decrease the number of relative roles and therefore increase efficiency of actions influence on well-being. The resulting recommendations allowed for stronger advocacy of an integrated decision approach as the four action categories were evaluated together rather than independently.

As with all network tools resolution is a critical feature of overall organization that must be considered in developing and using network indices in any specific context. This is most readily apparent in ecological networks like food webs, which are sensitive to resolution choices in node definition that range from single nodes for each functional group (e.g., primary producers) down to species specific nodes that can result in large increases in complexity (Zorach and Ulanowicz, 2003). In the eco-decisional network this is reflected in the wide range of network indices resulting from changes in network size (i.e., node count; Figure 4). Yet, the choice to use Action categories, which allow for a range of specific actions to be reflected in a single node and the use of stakeholder input to define ‘Action category’ and ‘Service’ nodes added important structure to the network definition and greatly reduces the observed variability. Further, the application of the Human Well-Being Index (Summers et al., 2016), which was built on specific well-being domains, provides both conceptual and analytical structure to the desired endpoints for decision making. Connectivity and Realized Roles are tied to how we define AC, as well as influence of AC on services, which were defined through input across multiple communities and the identification of common ground between communities.

Networks help visualize relationships so that actions chosen do not contradict each other (tradeoffs) or accomplish similar things (redundancy in perceived roles vs. realized roles), and these trade-offs can be examined and optimized as a comparison of specific decision pathways in the context of the other options. These features exploit the theory of network analysis in a novel way that is accessible and can be tied to community input. Community-specific eco-decisional network tools are designed to aid community decision makers of all forms make the shift towards a common goal of improving stakeholder well-being. Eco-decisional well-being networks can also be adapted to specific community goals and compared across community types (Fulford et al., 2015) to better inform integrated decision making. It is also important that networks provide a repeatable framework. Lau et al. (2017) highlighted the need for reproducibility and meaningful benchmarks for comparison, and our network approach was designed to be highly transferable across communities and integrated across issues of interest. Future work will involve development of visualization tools and more quantitative pathway analysis so that these features of networks can also be applied to specific community decision making.

## Supplementary Material

SI

## Figures and Tables

**TABLE 1:** Summary of nodes by category.

Action categories (n=29)	Service categories (n=22)	Domains of human wellbeing (n=9)
Access to natural resources	Capital Investment	Connection to nature
Preserve existence value	Production	Cultural fulfillment
Preserve sense of identity/place	Innovation	Education
Access to arts/music	Employment	Health
Access to children’s programs	Re-Distribution	Leisure time
Diversity of population	Consumption	Living standards
Access to food and cuisine	Finance	Safety and security Social cohesion
Access to government (input)	Greenspace	**HWBI** *
Knowledge of history	Air Quality	
Options for philanthropy	Water Quality	
Access to education	Food, Fiber and Fuel Provisioning	
Diversity of education content	Water Quantity	
Increase non-traditional options	Activism	
Access to healthcare	Emergency Preparedness	
Focus on youth education	Public works	
Access health education	Communication	
Support active lifestyle	Community and Faith-based Initiatives	
Access to basic standard of living	Education Services	
Preserve/promote local culture	Labor	
Support urban revitalization	Healthcare	
Support agriculture	Justice	
Diversity of jobs	Family Services	
Support economic development		
Healthy natural and built environment		
Access to housing/lifestyle options		
Promote community atmosphere		
Support faith institutions		
Support population stability/retention		
Access to transportation options		

(*) See Figure 1 for node ordering in decisional networks and Supplementary Materials for formal node descriptions as well as full link details. Items in same row are not necessarily linked in the network. The HWB nodes include eight domain nodes and one node for the overall HWBI.

## Data Availability

The original contributions presented in the study are publicly available. This data can be found here: EPA ScienceHub, https://catalog.data.gov/harvest/epa-sciencehub.
