# Supplementary material for: Eco-decisional well-being networks as a tool for community decision support
Source: Front Ecol Evol. Author manuscript; Available in PMC 2025 Apr 18. (PMC11457163; doi:10.3389/fevo.2024.1210154)
Supplement: SI [file NIHMS1988769-supplement-SI.pdf]

*R.S. Fulford and E. Paulukonis

*corresponding author: Fulford.Richard@epa.gov

Supplementary data on nodes, links and link weights are provided for the community-specific version of the eco-decisional network. Determination of nodes was based on three categories: Action categories, ecosystem services, and domains of human well-being. Links and weights between nodes were determined based on community input acquired through direct engagement as well as keyword analysis of community planning documents. See paper text for details.

Development of edge weights from stake holder information

Numerical weights used in the network analysis were developed from two data sources independently for three edge types (Action Category-Service, Service to Wellbeing Domain, Domain to Human wellbeing Index). The two data sources were data from direct stakeholder engagement in nine communities (Community comparison Report Chapter 4; https://cfpub.epa.gov/si/si_public_record_report.cfm?Lab=NHEERL&direntryid=330853) and a keyword analysis of six additional communities based on published strategic planning documents (Fulford, Krauss, Yee and Russell 2017). The keyword analysis was used primarily for the first edge type (Action category to Service) as these connections deal specifically with chosen actions and their direct benefits. Stake holder engagement provided data for informing weights on all three edge types with specific data collected to weight Domain to HWBI weights based on stakeholder priorities. Full details can be found in the cited reports and are summarized here.

Action category to Service (AC-S) – Action category nodes (n=29) are potential actions taken and are therefore the direct outcome of a decision. Action categories were identified in planning documents or in stake holder discussion during engagement events by specific mention and then linked to Service nodes (n=22) through further discussion of desire outcomes from these actions. A mapping exercise was used (Figure S1) to link Actions to Services and then weights are assigned to the established links based on number of times the selected link was mentioned across all engagement events and community documents analyzed. Action category to Service link weights were based on direct counts normalized (0-1) to the maximums count across all AC-S links identified.

Service to Domain of Wellbeing (S-D) – Links between Service nodes and Domains (n=8) of wellbeing nodes represent how a pathway for changes in service production to be delivered to human beneficiaries via a change in some aspect of human wellbeing. Service to domain weights were based on a combination of the analytical analysis of Service influence on wellbeing (cite Summers) and the mapping exercise conducted during engagement events (Figure S1, cite report). Services mentioned by stakeholders were ranked based on a count of number of mentions across all nine engagement events normalized (0-1) to the maximum count for a specific service. Service-Domain links considered in the

numerical analysis were similarly normalized and combined with engagement results with a simple average of the two normalized values.

Domains of Wellbeing to Human Wellbeing Index (D-HWBI) – Links between the Domains of human wellbeing and the Human Wellbeing Index value (n=1) represent the calculated cumulative outcome of decisions to overall wellbeing. Weights for the D-HWBI links were estimated from three separate ranking exercise conducted during all engagement events. First ranking among theses eight links were estimated from the mapping exercise and the number of times each D-HWBI link was mentioned across all engagement events. The second ranking was a group dot voting exercise conducted separately for each engagement event in which participants were asked to 'value the eight domains of Wellbeing by placing stickers on a large list of the domains placed on a wall. Each participant was given eight stickers and could place them however they wished or not at all. Link weight for dot voting was a count of 'dots' placed on each domain within each engagement events and then averaged across all engagement events. The third ranking was an anonymous ranking exercise in which all participants were asked to rank the domains from 1-8 with 1 being the most important. Median rank for each Domain was recorded for each engagement events and overall rank was median rank across all engagement events. All D-HWBI rankings were normalized (0-1) within type and combined across types with a simple average.

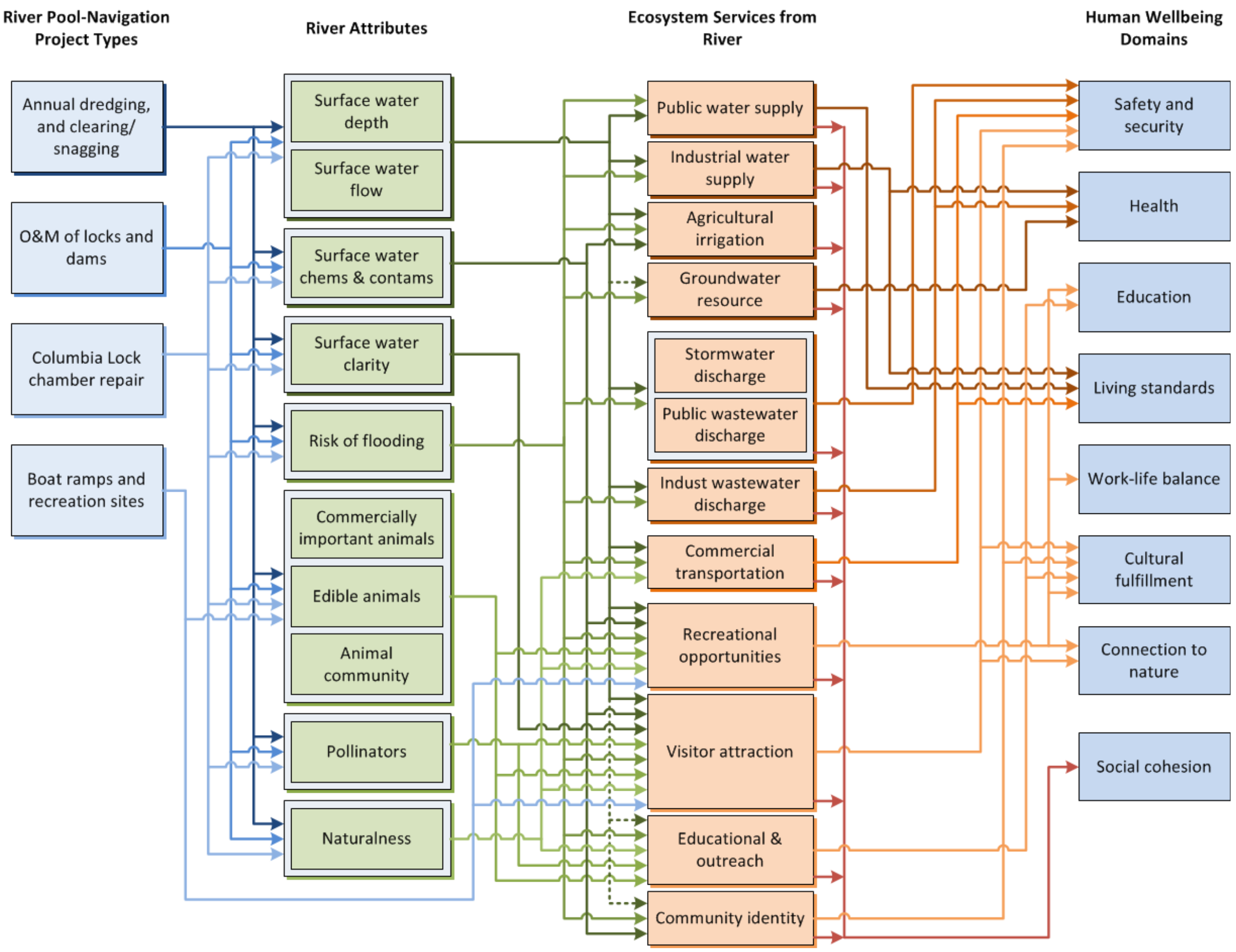

Figure S1 - Concept map describing links between project types, river attributes, ecosystem services, and domains of human well-being impacted by river pool navigation projects on the Ouachita River. Action categories in this case were combined project types and river attributes affected. This concept map is the result of information collected during stakeholder Workshop 2 held in West Monroe, LA (Deeper Look at the Ouachita River; https://cfpub.epa.gov/si/si_public_file_download.cfm?p_download_id=542172&Lab=CEMM).

Supplementary Table 1. Summary of links and weights derived for the community specific eco-decisional network. Links are organized by level based on the source node but ordered numerically within level by target node. Only links with a non-zero weight are listed. See text for details on derivation of link weights.

From	To	Weight		From	To	Weight		From	To	Weight
D1	HWBI	0.79		S1	D1	0.26		AC1	S1	0.67
D2	HWBI	0.81		S2	D1	0.84		AC4	S1	0.67
D3	HWBI	0.91		S5	D1	0.60		AC6	S1	0.07
D4	HWBI	0.88		S6	D1	0.84		AC7	S1	0.40
D5	HWBI	0.81		S7	D1	0.60		AC17	S1	0.20
D6	HWBI	0.87		S8	D1	0.55		AC19	S1	0.40
D7	HWBI	0.94		S9	D1	1.00		AC20	S1	0.67
D8	HWBI	1.00		S10	D1	0.72		AC22	S1	0.33
				S11	D1	0.72		AC23	S1	1.00
				S13	D1	0.19		AC25	S1	0.67
				S14	D1	0.00		AC29	S1	0.67
				S15	D1	0.88		AC1	S2	0.67
				S16	D1	0.77		AC2	S2	0.33
				S17	D1	0.88		AC3	S2	0.13
				S18	D1	0.77		AC4	S2	0.33
				S19	D1	0.13		AC5	S2	0.20
				S20	D1	0.67		AC6	S2	0.20
				S21	D1	0.11		AC7	S2	0.20
				S1	D2	0.06		AC8	S2	0.33
				S2	D2	0.01		AC9	S2	0.20
				S3	D2	0.09		AC10	S2	0.67
				S8	D2	0.44		AC11	S2	0.67
				S9	D2	0.01		AC12	S2	0.67
				S12	D2	0.00		AC13	S2	0.07
				S14	D2	0.08		AC14	S2	0.67
				S15	D2	0.06		AC15	S2	0.33
				S16	D2	0.55		AC16	S2	0.33
				S17	D2	1.00		AC17	S2	0.07
				S18	D2	0.13		AC18	S2	0.67
				S19	D2	0.06		AC19	S2	0.20
				S22	D2	0.13		AC20	S2	1.00
				S1	D3	0.46		AC21	S2	1.00
				S2	D3	0.00		AC22	S2	0.67
				S4	D3	0.46		AC23	S2	0.33
				S5	D3	0.46		AC24	S2	0.33
				S6	D3	0.08		AC25	S2	0.33

From	To	Weight		From	To	Weight		From	To	Weight
				S8	D3	0.46		AC26	S2	0.07
				S13	D3	0.71		AC27	S2	0.20
				S15	D3	0.38		AC28	S2	0.40
				S17	D3	0.64		AC29	S2	0.33
				S18	D3	1.00		AC10	S3	0.67
				S21	D3	0.03		AC11	S3	0.33
				S22	D3	0.56		AC12	S3	0.67
				S1	D4	0.26		AC23	S3	0.33
				S3	D4	0.28		AC7	S4	0.60
				S4	D4	0.26		AC11	S4	0.67
				S5	D4	0.26		AC12	S4	0.67
				S6	D4	0.26		AC14	S4	0.33
				S8	D4	0.64		AC18	S4	1.00
				S9	D4	0.64		AC21	S4	0.67
				S10	D4	0.64		AC22	S4	1.00
				S11	D4	0.64		AC23	S4	0.33
				S12	D4	0.10		AC26	S4	0.07
				S13	D4	0.17		AC28	S4	0.20
				S14	D4	0.17		AC6	S5	0.13
				S15	D4	0.26		AC14	S5	0.33
				S16	D4	0.51		AC7	S6	0.40
				S17	D4	0.17		AC14	S6	0.33
				S18	D4	0.17		AC18	S6	0.67
				S19	D4	0.28		AC20	S6	0.67
				S20	D4	1.00		AC21	S6	0.33
				S21	D4	0.40		AC10	S7	1.00
				S22	D4	0.22		AC23	S7	0.67
				S1	D5	0.25		AC1	S8	1.00
				S3	D5	0.33		AC2	S8	1.00
				S4	D5	0.23		AC11	S8	0.33
				S6	D5	0.20		AC15	S8	0.33
				S7	D5	0.50		AC16	S8	0.33
				S8	D5	0.52		AC17	S8	0.13
				S10	D5	0.40		AC19	S8	0.20
				S11	D5	0.85		AC20	S8	0.33
				S12	D5	0.00		AC24	S8	0.67
				S13	D5	1.00		AC26	S8	0.07
				S15	D5	0.78		AC28	S8	0.20
				S16	D5	0.50		AC2	S9	1.00
				S17	D5	0.30		AC24	S9	1.00

From	To	Weight		From	To	Weight		From	To	Weight
				S18	D5	0.11		AC2	S10	1.00
				S20	D5	0.52		AC24	S10	1.00
				S21	D5	0.56		AC14	S11	0.33
				S1	D6	0.37		AC24	S11	0.67
				S2	D6	0.38		AC24	S12	0.67
				S4	D6	0.56		AC3	S13	0.07
				S5	D6	0.57		AC11	S13	0.33
				S6	D6	0.57		AC19	S13	0.40
				S7	D6	0.00		AC26	S13	0.40
				S8	D6	0.73		AC27	S13	0.40
				S10	D6	0.38		AC8	S14	0.33
				S11	D6	0.56		AC10	S14	0.33
				S12	D6	0.54		AC14	S14	0.67
				S13	D6	1.00		AC26	S14	0.40
				S15	D6	0.18		AC28	S14	0.20
				S16	D6	0.56		AC29	S14	0.33
				S17	D6	0.57		AC1	S15	0.33
				S18	D6	0.57		AC2	S15	0.33
				S20	D6	0.57		AC5	S15	0.20
				S21	D6	0.13		AC6	S15	0.07
				S1	D7	0.26		AC8	S15	1.00
				S7	D7	0.08		AC9	S15	0.20
				S8	D7	0.08		AC11	S15	0.33
				S9	D7	0.08		AC12	S15	0.33
				S10	D7	0.00		AC13	S15	0.07
				S11	D7	0.63		AC15	S15	0.33
				S12	D7	0.43		AC16	S15	0.33
				S13	D7	0.72		AC17	S15	0.13
				S14	D7	0.08		AC19	S15	0.20
				S15	D7	0.00		AC20	S15	0.33
				S17	D7	0.63		AC24	S15	0.33
				S18	D7	0.26		AC25	S15	0.67
				S19	D7	1.00		AC26	S15	0.07
				S20	D7	0.45		AC28	S15	0.20
				S21	D7	0.45		AC29	S15	1.00
				S22	D7	0.45		AC3	S16	0.20
				S1	D8	0.20		AC8	S16	0.67
				S2	D8	0.00		AC2	S17	0.67
				S4	D8	0.15		AC3	S17	0.20
				S5	D8	0.70		AC4	S17	1.00

From	To	Weight		From	To	Weight		From	To	Weight
				S6	D8	0.10		AC6	S17	0.13
				S8	D8	0.71		AC8	S17	0.33
				S9	D8	0.18		AC9	S17	0.60
				S10	D8	0.15		AC10	S17	0.33
				S15	D8	0.18		AC13	S17	0.20
				S16	D8	0.23		AC19	S17	0.60
				S17	D8	1.00		AC25	S17	1.00
				S18	D8	0.12		AC26	S17	0.20
				S19	D8	0.21		AC27	S17	0.60
				S20	D8	0.20		AC28	S17	0.60
				S21	D8	0.62		AC5	S18	0.60
				S22	D8	0.23		AC9	S18	0.40
								AC11	S18	1.00
								AC12	S18	1.00
								AC13	S18	0.13
								AC15	S18	1.00
								AC16	S18	1.00
								AC18	S18	0.33
								AC26	S18	0.07
								AC28	S18	0.20
								AC8	S19	0.33
								AC14	S20	1.00
								AC16	S20	0.67
								AC18	S20	0.33
								AC25	S20	0.33
								AC28	S20	0.20
								AC18	S21	0.33
								AC20	S21	0.33
								AC25	S21	0.33
								AC26	S21	0.07
								AC5	S22	0.40
								AC8	S22	0.33
								AC10	S22	0.33
								AC15	S22	0.67
								AC27	S22	0.40
